# The neuroprotective effects of targeting key factors of neuronal cell death in neurodegenerative diseases: The role of ER stress, oxidative stress, and neuroinflammation

**DOI:** 10.3389/fncel.2023.1105247

**Published:** 2023-03-06

**Authors:** Mohammad Sobhan Karvandi, Farzam Sheikhzadeh Hesari, Amir Reza Aref, Majid Mahdavi

**Affiliations:** ^1^Department of Cell and Molecular Sciences, Faculty of Biological Sciences, Kharazmi University, Tehran, Iran; ^2^Department of Animal Biology, Faculty of Natural Sciences, University of Tabriz, Tabriz, Iran; ^3^Department of Medical Oncology, Belfer Center for Applied Cancer Science, Dana-Farber Cancer Institute and Harvard Medical School, Boston, MA, United States; ^4^Institute of Biochemistry and Biophysics, University of Tehran, Tehran, Iran

**Keywords:** neurodegenerative diseases, cell death, ER stress, UPR – unfolded protein response, oxidative stress, ROS, neuroinflammation

## Abstract

Neuronal loss is one of the striking causes of various central nervous system (CNS) disorders, including major neurodegenerative diseases, such as Alzheimer’s disease (AD), Parkinson’s disease (PD), Huntington’s disease (HD), and Amyotrophic lateral sclerosis (ALS). Although these diseases have different features and clinical manifestations, they share some common mechanisms of disease pathology. Progressive regional loss of neurons in patients is responsible for motor, memory, and cognitive dysfunctions, leading to disabilities and death. Neuronal cell death in neurodegenerative diseases is linked to various pathways and conditions. Protein misfolding and aggregation, mitochondrial dysfunction, generation of reactive oxygen species (ROS), and activation of the innate immune response are the most critical hallmarks of most common neurodegenerative diseases. Thus, endoplasmic reticulum (ER) stress, oxidative stress, and neuroinflammation are the major pathological factors of neuronal cell death. Even though the exact mechanisms are not fully discovered, the notable role of mentioned factors in neuronal loss is well known. On this basis, researchers have been prompted to investigate the neuroprotective effects of targeting underlying pathways to determine a promising therapeutic approach to disease treatment. This review provides an overview of the role of ER stress, oxidative stress, and neuroinflammation in neuronal cell death, mainly discussing the neuroprotective effects of targeting pathways or molecules involved in these pathological factors.

## Introduction

Neurodegenerative diseases are nervous system disorders in which millions of people, especially the elderly, are being affected worldwide. The rising prevalence of these diseases has put the world with a serious challenge (Bloem et al., 2021; Milošević et al., 2021). Despite the developments in this field of study and advancements in pharmacological aspects, there is not a promising drug to consummately cure neurodegenerative diseases yet (Pohl and Kong Thoo Lin, 2018). However, there are still so many studies to alleviate disease symptoms and extend life span (Breijyeh and Karaman, 2020). Neurodegenerative diseases are mostly characterized by toxic protein aggregates with abnormal conformation within neurons or neuroglia, leading to memory, cognitive, and/or movement disorders (Dugger and Dickson, 2017). These diseases include a wide range of neurological disorders, but the major types are Alzheimer’s disease (AD), Parkinson’s disease (PD), Huntington’s disease (HD), and amyotrophic lateral sclerosis (ALS; Lezi and Swerdlow, 2012). Protein misfolding and accumulation of amyloid-β (Aβ) and phosphorylated Tau is the major pathological feature in AD, as well as α-synuclein in PD, and mutant superoxide dismutase 1 (mSOD1) in ALS (Ghemrawi and Khair, 2020). Clinical manifestations in these kinds of diseases mainly occur as a consequence of neuron dysfunction or neuronal cell death (Andreone et al., 2020). Besides apoptosis, depending on the conditions, other different types of cell deaths, such as ferroptosis, necroptosis, and parthanatos are also possible to affect cell fidelity (Wang et al., 2018; Ferrada et al., 2020; Reichert et al., 2020; David et al., 2022; Mangalmurti and Lukens, 2022). The mitochondria and endoplasmic reticulum (ER) play crucial role in the occurrence of neuronal cell death among many other organelles (Gorman et al., 2012; Johnson et al., 2021; Markovinovic et al., 2022). Mitochondria are dynamic organelles that participate in producing energy and maintaining cellular redox balance, among many other functions (Johri and Beal, 2012). Therefore, mitochondrial dysfunction, including excessive reactive oxygen species (ROS) production, mitochondrial calcium overload, loss of the mitochondrial membrane potential leading to release of apoptosis-inducing factor (AIF), and other pro-apoptotic factors could lead to caspase activation and cell death (Culmsee and Plesnila, 2006; Kaminskyy and Zhivotovsky, 2014; Hoffmann et al., 2021). Evidence also reveals that mitochondrial DNA (mtDNA) mutations are present in patients with neurodegeneration (Johri and Beal, 2012). Also, aberrant ROS production and imbalance in antioxidant activity could influence mitochondria and impair mitochondria’s function, leading cells to death (Angelova and Abramov, 2018; Doroudian et al., 2021), which is explained in the following sections. Of note, ROS can also contribute to the production of protein aggregates and exacerbate disease pathology (Van Dam and Dansen, 2020).

On the other hand, the ER is a large and dynamic organelle responsible for protein folding and maturation. Once a protein folds with an abnormal conformation, the misfolded protein enters the ER-associated degradation (ERAD) pathway to prevent the following plausible detrimental effects of the protein (Schwarz and Blower, 2016). Aberrant misfolded proteins or aggregates can potentially trigger the process “Unfolded Protein Response” (UPR) to attenuate ER stress or initiate apoptosis pathways (Schwarz and Blower, 2016). UPR has three signaling arms, including IRE1-α, PERK, and ATF6, which are highly conserved pathways (Shi et al., 2022). However, toxic protein aggregates may also undergo degradation by lysosomes (i.e., autophagy), to ameliorate disease progression (Djajadikerta et al., 2020). Autophagy is able to activate or inhibit the apoptosis signaling to maintain intracellular balance or induce neuronal cell death (Gupta R. et al., 2021). All three UPR arms, Ca^2+^ release, and oxidative stress can directly or indirectly activate autophagy induction (Andhavarapu et al., 2019; Ramirez-Moreno et al., 2019; Ren et al., 2021). Although protein aggregates are the key reasons for the pathology of neurodegenerative diseases, other factors, including activation of glutamate ionotropic receptors, excitotoxicity from dysregulation of neuronal calcium homeostasis, dysfunction of lysosomes, aberrant cell-cycle re-entry, and impairments in axonal transport and synaptic function can also contribute to neuronal injury or death in various neurodegenerative diseases such as AD and PD (Emerit et al., 2004; Fricker et al., 2018; Sushma and Mondal, 2019; Behl et al., 2021; Hoffmann et al., 2021). In addition, increased levels of inflammatory factors in the serum and brain tissue, known as neuroinflammation, participates in the pathophysiology of neurodegenerative diseases (Calsolaro and Edison, 2016). Emerging evidence indicates that neuroinflammation can be the cause and consequence of both ER stress and oxidative stress (Salminen et al., 2009; Sochocka et al., 2013; Pintado et al., 2017). A neurotoxic microenvironment caused by the activation of microglial cells and release of cytotoxic inflammatory factors in the CNS can affect cell fidelity and induce neuronal cell death (Behl et al., 2021; Wu and Zou, 2022). This can be carried out by triggering pyroptosis, an inflammasome-mediated type of cell death (Kovacs and Miao, 2017). However, the undeniable contribution of age, genetics, and environmental factors in the disruption of neuronal homeostasis and subsequently neuronal cell death cannot be discounted (Bejanin et al., 2017).

There have been clinical trials targeting neuropathological hallmarks of neurodegenerative diseases, investigating glucagon-like peptide-1 receptor (GLP-1R) agonists, monoclonal antibodies against toxic protein aggregates, antioxidant agents, beta-secretase (BACE1) inhibitors and other receptor inhibitors such as 5HT-6 serotonin receptor inhibitor (Table 1, Hung and Fu, 2017). The results were controversial, as there was no evidence of beneficial effect on patients’ cognitive and functional status in most trials; while in some cases the condition of patients who received drugs worsened, compared with those who received placebo (Table 1, Egan et al., 2019). These results indicate that novel agents with different features must be studied and trialled. Given the complex interplay of ER stress, oxidative stress, and neuroinflammation in the pathology of most neurodegenerative diseases, developments in the knowledge of underlying mechanisms may be crucial for researchers to propose a promising therapeutic strategy to achieve a more efficient treatment for neurodegenerative diseases.

**Table 1:** Clinical trials associated with neurodegenerative diseases.

**Trial Identification**	**Drug used**	**Drug description**	**Delivery route**	**Disease**	**Phase**	**Status**	**Results**
**NCT03659682**	Semaglutide	GLP-1R agonistprevents neurons from apoptosis, alleviates oxidative stress and neuroinflammation (Chen et al., 2023)	Subcutaneous	PD	II	Not yet recruiting	NA
**NCT03439943**	Lixisenatide	GLP-1R agonist prevents neurons from apoptosis, alleviates oxidative stress and neuroinflammation (Chen et al., 2023)	Subcutaneous	PD	II	Unknown	NA
**NCT04305002**	Exenatide	GLP-1R agonist prevents neurons from apoptosis, alleviates oxidative stress and neuroinflammation (Chen et al., 2023)	Subcutaneous	PD	II	Active, not recruiting	NA
**NCT04232969**	Bydureon (Exenatide)	GLP-1R agonist prevents neurons from apoptosis, alleviates oxidative stress and neuroinflammation (Chen et al., 2023)	Subcutaneous	PD	III	Active, not recruiting	NA
**NCT04154072**	NLY01	a pegylated form of exenatide (Lv et al., 2021)	Subcutaneous	PD	II	Active, not recruiting	NA
**NCT04269642**	PT320	sustained-release Exenatide (Li et al., 2019)	Subcutaneous	PD	II	Active, not recruiting	NA
**NCT04777409**	Semaglutide	GLP-1R agonist prevents neurons from apoptosis, alleviates oxidative stress and neuroinflammation (Chen et al., 2023)	Oral	AD	III	Recruiting	NA
**NCT02953665**	Liraglutide	GLP-1R agonist prevents neurons from apoptosis, alleviates oxidative stress and neuroinflammation (Chen et al., 2023)	Subcutaneous	PD	II	Completed	NA
**NCT00004731**	Coenzyme Q10	An antioxidant involved in electron transport chain (Gherardi et al., 2022)	NA	PD	II	Completed	NA
**NCT00608881**	Coenzyme Q10	An antioxidant involved in electron transport chain (Gherardi et al., 2022)	Oral	HD	III	Terminated	CoQ had no effect on avoiding functional decline in HD patients (Mcgarry et al., 2017)
**NCT01892176**	Coenzyme Q10	An antioxidant involved in electron transport chain (Gherardi et al., 2022)	Oral	PD	II and III	Completed	NA
**NCT00243932**	Coenzyme Q10	An antioxidant involved in electron transport chain (Gherardi et al., 2022)	Oral	ALS	II	Completed	Showed insufficient promise to warrant phase III testing (Kaufmann et al., 2009)
**NCT00740714**	Coenzyme Q10 with VitE	VitE: a fat-soluble antioxidant (Blaner et al., 2021)	Oral	PD	III	Terminated	No evidence of benefit
**NCT00076492**	CoQ10 and GPI 1485	GPI 1485: a neuroimmunophilin ligand (Poulter et al., 2004)	NA	PD	II	Completed	NA
**NCT03514875**	MitoQ	A mitochondrial reactive oxygen species scavenger (Piscianz et al., 2021)	Oral	AD	NA	Withdrawn	NA
**NCT00329056**	MitoQ	A mitochondrial reactive oxygen species scavenger (Piscianz et al., 2021)	Oral	PD	II	Completed	NA
**NCT04777331**	Prasinezumab	Humanized monoclonal antibody against aggregated α-synuclein (Pagano et al., 2022)	Intravenous (IV) infusion	PD	II	Recruiting	NA
**NCT03114657**	Crenezumab	Monoclonal antibody against Aβ (Avgerinos et al., 2021)	Intravenous (IV) infusion	AD	III	Terminated	Could not reduce clinical decline in participants with early AD (Ostrowitzki et al., 2022)
**NCT03491150**	Crenezumab	Monoclonal antibody against Aβ (Avgerinos et al., 2021)	Intravenous (IV) infusion	AD	III	Terminated	Crenezumab was unlikely to meet its primary endpoint
**NCT00676143**	Bapineuzumab	Monoclonal antibody against Aβ (Avgerinos et al., 2021)	Intravenous (IV) infusion	AD	III	Terminated	Phase 3 studies showed no clinical benefit
**NCT00606476**	Bapineuzumab	Monoclonal antibody against Aβ (Avgerinos et al., 2021)	Intravenous (IV) infusion	AD	II	Terminated	NA
**NCT01656525**	Gantenerumab	Monoclonal antibody against Aβ (Avgerinos et al., 2021)	Subcutaneous	AD	I	Completed	NA
**NCT02051608**	Gantenerumab	Monoclonal antibody against Aβ (Avgerinos et al., 2021)	Subcutaneous	AD	III	Completed	Gantenerumab doses up to 1200 mg resulted in robust amyloid-β plaque removal at 2 years (Klein et al., 2019)
**NCT04374253**	Gantenerumab	Monoclonal antibody against Aβ (Avgerinos et al., 2021)	Subcutaneous	AD	III	Active, not recruiting	NA
**NCT03444870**	Gantenerumab	Monoclonal antibody against Aβ (Avgerinos et al., 2021)	Subcutaneous	AD	III	Active, not recruiting	NA
**NCT03443973**	Gantenerumab	Monoclonal antibody against Aβ (Avgerinos et al., 2021)	Subcutaneous	AD	III	Active, not recruiting	NA
**NCT05310071**	Aducanumab	Monoclonal antibody against Aβ (Dhillon, 2021)	Intravenous (IV) infusion	AD	III	Recruiting	NA
**NCT03639987**	Aducanumab	Monoclonal antibody against Aβ (Dhillon, 2021)	Intravenous (IV) infusion	AD	II	Terminated	Study was discontinued based on futility analysis conducted on Phase III trials
**NCT05108922**	Aducanumab, Donanemab	Monoclonal antibody against Aβ (Decourt et al., 2021)	Intravenous (IV) infusion	AD	III	Active, not recruiting	NA
**NCT03582137**	Cannabidiol	A major constituent of *Cannabis sativa L.* (Karimi-Haghighi et al., 2022)	Oral	PD	II	Completed	NA
**NCT01502046**	Sativex	Contains Tetrahydrocannabinol and Cannabidiol in a 1:1 molecular ratio (Cristino et al., 2020)	Oromucosal Spray	HD	II	Completed	No significant molecular effects were detected on the biomarker analysis No significant symptomatic effects were detected at the prescribed dosage and for a 12-week period (López-Sendón Moreno et al., 2016)
**NCT04075435**	High CBD/low THC sublingual solution	CBD: Cannabidiol THC: Tetrahydrocannabinol	Sublingual	AD	Early phase I	Recruiting	NA
**NCT02783573**	Lanabecestat (AZD3293)	BACE1 inhibitor (Patel et al., 2022)	Oral	AD	III	Terminated	Did not slow cognitive or functional decline (Wessels et al., 2020)
**NCT02245737**	Lanabecestat (AZD3293)	BACE1 inhibitor (Patel et al., 2022)	Oral	AD	II and III	Terminated	Did not slow cognitive or functional decline (Wessels et al., 2020)
**NCT02956486**	Elenbecestat (E2609)	BACE1 inhibitor (Patel et al., 2022)	Oral	AD	III	Terminated	No evidence of potential efficacy, and the adverse event profile of E2609 being worse than placebo
**NCT01600859**	Elenbecestat (E2609)	BACE1 inhibitor (Patel et al., 2022)	Oral	AD	I	Completed	NA
**NCT01496170**	Verubecestat (MK-8931)	BACE1 inhibitor (Patel et al., 2022)	Oral	AD	I	Completed	NA
**NCT01739348**	Verubecestat (MK-8931)	BACE1 inhibitor (Patel et al., 2022)	Oral	AD	II and III	Terminated	Did not reduce cognitive or functional decline in patients with mild-to-moderate Alzheimer’s disease (Egan et al., 2018)
**NCT01953601**	Verubecestat (MK-8931)	BACE1 inhibitor (Patel et al., 2022)	Oral	AD	III	Terminated	Cognition and daily function were worse among patients who received verubecestat than among those who received placebo Did not improve clinical ratings of dementia among patients with prodromal Alzheimer’s disease (Egan et al., 2019)
**NCT01689246**	TRx0237	Tau aggregation inhibitor (Hung and Fu, 2017)	Oral	AD	III	Completed	No evidence of benefits for patients with mild to moderate Alzheimer’s disease (Gauthier et al., 2016)
**NCT03539380**	TRx0237	Tau aggregation inhibitor (Hung and Fu, 2017)	NA	AD	NA	Available	NA
**NCT02585934**	Intepirdine (RVT-101) and donepezil	Intepirdine: 5HT-6 serotonin receptor inhibitor (Hung and Fu, 2017) Donepezil: acetylcholinesterase inhibitor (Marucci et al., 2021)	Oral	AD	III	Completed	Did not produce statistical improvement over placebo on cognition or activities of daily living in mild-to-moderate AD dementia patients (Lang et al., 2021)

## ER stress-induced cell death in neurodegenerative diseases

### Mechanism of ER stress-induced apoptotic cell death

The ER is known as an organelle involved in protein maturation and folding (Read and Schröder, 2021). Toxic protein aggregates in neurodegenerative diseases, pathogen-associated molecular patterns (PAMPs), danger-associated molecular patterns (DAMPs), ROS, and reactive nitrogen species (RNS), can disrupt protein folding processes in the ER lumen, leading to ER stress (Zhang and Kaufman, 2008). In the condition of ER stress, the aggregation of unfolded or misfolded proteins within the ER lumen of neurons and neuroglia leads to failure of ER in maintaining protein homeostasis through UPR and ERAD. For instance, accumulation of tau protein in AD can affect essential components of ERAD and block this pathway, leading to the accumulation of more misfolded proteins in the ER lumen (Hetz and Saxena, 2017; Ghemrawi and Khair, 2020). Subsequently, UPR-dependent inflammation and apoptotic pathways are induced, resulting in neuronal cell death (Sprenkle et al., 2017; Ghemrawi and Khair, 2020). The ER stress can also be induced by ER Ca^2+^ dysregulation, impairments in vesicular trafficking, or any defects in UPR components (Cooper et al., 2006; Sprenkle et al., 2017). PKR-like ER kinase (PERK), inositol-requiring transmembrane kinase/endoribonuclease 1 α (IRE1α), and activating transcription factor 6 (ATF6) are three vital sensor proteins that are involved in UPR regulation (Ghemrawi and Khair, 2020). Under normal conditions, these proteins are inactive due to association with ER chaperone proteins such as Immunoglobulin binding protein (BiP) or 78 kDa glucose-regulated protein (GRP78), which are members of heat shock protein families (Halperin et al., 2014).

**Figure 1:**
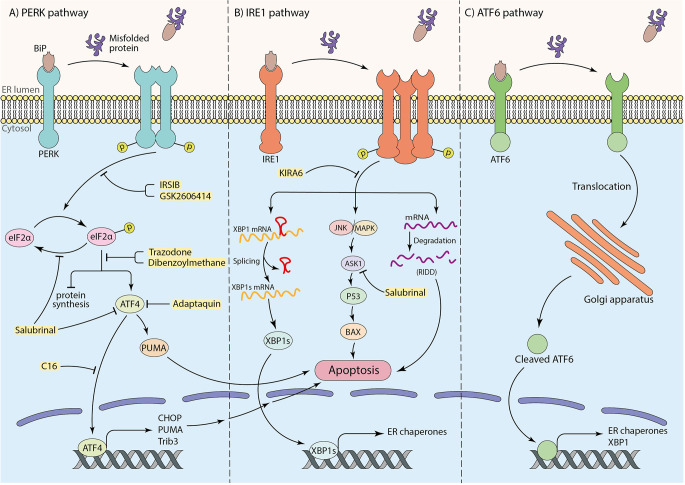
The role of three arms of UPR in inducing apoptosis and the neuroprotective effects of particular inhibitors (shown in yellow box). **(A)** The PERK pathway: interaction of substrate binding domain of BiP with misfolded or aggregated proteins leads to BiP dissociation, dimerization, and autophosphorylation of PERK, which further causes eIF2α phosphorylation. Phosphorylated eIF2α induces cell death by transcription of apoptotic factors by means of ATF4 transcription factor as well as inhibition of protein synthesis. **(B)** The IRE1 pathway: after dissociation of BiP from IRE1 receptor by misfolded or aggregated proteins in the ER lumen, IRE1 undergoes oligomerization and autophosphorylation. This results in mRNA degradation termed “regulated IRE1alpha-dependent decay” (RIDD) and inducing apoptotic factors by initiating JNK/MAPK cascade. To mitigate ER stress, the IRE1 pathway also leads to XBP1 mRNA splicing to transcript ER chaperones to improve ER machinery. **(C)** The ATF6 pathway: translocation of ATF6 to the Golgi apparatus as a result of BiP dissociation, and the proteolysis of ATF6 in Golgi brings out an activated ATF6 transcription factor to transcript ER chaperones and XBP1 for ER machinery.

Under ER stress conditions, the misfolded proteins interact with the substrate binding domain of BiP. Consequently, BiP is released and leads to dimerization and auto-phosphorylation of PERK, as well as intramembrane proteolysis of ATF6 and phosphorylation of IRE1α. Subsequently, the UPR cascade activates to maintain protein homeostasis (Ghemrawi and Khair, 2020). To elaborate, the phosphorylation of the alpha subunit of eukaryotic translation initiation factor (eIF2α) followed by activation of PERK occurs through BiP dissociation. This process inhibits protein synthesis to prevent overload of proteins in the ER lumen (Hetz and Saxena, 2017; Almeida et al., 2022), therefore attempting to restore protein homeostasis (Da Silva et al., 2020). Besides, under prolonged ER stress conditions and failure in the UPR mechanism, p-eIF2α promotes activating transcription factor 4 (ATF4) translation, which enhances up-regulation of pro-apoptotic factors, including CHOP (also known as GADD153; Ghemrawi and Khair, 2020). Eventually, down-regulation of anti-apoptotic Bcl-2 family makes neurons more susceptible to death (Doyle et al., 2011; Hetz and Saxena, 2017; Da Silva et al., 2020; Figure 1A). Moreover, it has been claimed that TRB3 genes, GADD34, death receptor 5 (DR5), ER oxidase 1 (ERO1), and other apoptotic molecules can potentially receive apoptosis signals from CHOP and induce cell death (Taalab et al., 2018; Da Silva et al., 2020). ATF4 also induces transcription of the p53-upregulated modulator of apoptosis (PUMA), which results in ER-stress-induced neuronal apoptosis (Galehdar et al., 2010). Interestingly, experiments have indicated that CHOP could not induce apoptosis in PUMA-deficient neurons, demonstrating the key role of PUMA in CHOP-induced neuronal apoptosis (Galehdar et al., 2010). Moreover, in IRE1-α signaling pathway, the second arm of UPR, after the release of BiP by aggregated proteins, IRE1-α undergoes oligomerization and auto-phosphorylation. p-IRE1α facilitates neuronal death by activation of the apoptotic-signaling kinase-1 (ASK1) and other apoptotic factors as a result of c-Jun N-terminal kinase (JNK) and p38 mitogen-activated protein kinase (p38 MAPK) pathways activation (Ghemrawi and Khair, 2020). This pathway may induce p53 activation and lead to the upregulation of Bcl-2 associated X (BAX) protein, which triggers the release of cytochrome C (Cyt C) from the mitochondria to the cytosol and cause apoptotic neuronal cell death (Stefani et al., 2012). Furthermore, the RNase activity of IRE1α plays a critical role in splicing the mRNA coding for X-box binding protein 1 (XBP1) and increases the expression of genes involved in ER machinery, such as BiP (Lee et al., 2003; Hirota et al., 2006; Chen et al., 2022). Besides outlined functions, IRE1α participates in the degradation of some mRNAs and microRNAs, known as “regulated IRE1α-dependent decay” (RIDD; Hetz and Saxena, 2017; Figure 1B). Mutations in Presenilin 1 and 2 (PS1 and PS2), which are frequently involved in AD, can inhibit IRE1 and impair UPR, leading to AD pathology and neuronal cell death (Doyle et al., 2011). ATF6 is the third sensor protein of UPR cascades which is embedded in the ER membrane. By interaction of aggregated proteins with ATF6 in the ER lumen and release of BiP, ATF6 translocates to the Golgi apparatus and undergoes proteolysis. Subsequently, cleaved ATF6 induces transcription of ER chaperones and XBP1 in the nucleus and participates in protein homeostasis (Da Silva et al., 2020; Figure 1C).

### ER stress-associated alterations of apoptotic factors

In AD, Aβ can trigger ER stress, mitochondrial fragmentation, and neuronal death through ER Ca^2+^ release by ryanodine receptors (RyRs) and inositol triphosphate receptors (IP3R; Chami and Checler, 2020). Based on studies, overexpression of RyRs contributed to Ca^2+^ dysregulation in AD mouse models and cell lines. In addition, increase in IP3 receptor-mediated Ca^2+^ signaling was indicated in AD patients’ fibroblast cells (Callens et al., 2021). Aβ oligomer-dependent ER stress responses can subsequently activate different kinases which phosphorylate specific epitopes on tau leading to the development of neurofibrillary tangles (NFTs) and propagating AD pathology (Sprenkle et al., 2017). Aβ peptides can activate ASK1 and JNK pathways, which can subsequently mediate ER stress-induced apoptosis (Ghemrawi and Khair, 2020). Both ASK1 and JNK were reported to be upregulated in transgenic mouse brains and post-mortem AD samples, respectively (Galvan et al., 2007; Sbodio et al., 2019). It has been revealed that CHOP activation plays a crucial role in the triggering and progression of pathological hallmarks of AD. In agreement, CHOP and its downstream effectors, including caspase-12 and GADD34, are markedly upregulated in the brains of AD patients (Ghemrawi and Khair, 2020). In addition, phosphorylated forms of PERK and eIF2α were significantly increased in the hippocampal pyramidal cells and frontal cortex of AD patients (Stutzbach et al., 2013). The evidence also shows that ER chaperones, including BiP, are also upregulated in the cerebrospinal fluid (CSF) and AD brains (Ghemrawi and Khair, 2020).

Mutations in PARK7, a gene involved in familial PD, might activate ASK1-induced neuronal death in PD. This can be due to the dysfunction in protecting against the Daxx-ASK1 cell death axis, which plays a key role in the completion of signaling pathways from cell surface death receptors (Chang et al., 1998; Homma et al., 2009). In addition, upregulation of ER stress markers, such as GRP78, p-PERK, and p-eIF2α in dopaminergic (DA) neurons of post-mortem PD samples (Shi et al., 2022), demonstrate their function in initiating apoptosis pathways, which could cause serious clinical implications. According to evidence, upregulation in ER stress markers, including BiP and CHOP in post-mortem HD brains, may be associated with neuronal death in HD (Shi et al., 2022). Mutation in genes such as SOD1, a gene encoding Superoxide dismutase 1 (SOD1), can also induce ER stress in neurons in ALS and cause neuronal damages (Sprenkle et al., 2017). ALS-associated mutations in vesicle-associated membrane protein-associated protein B (VAPB) can physically interact with ATF6 and disturb its natural function (Hetz and Saxena, 2017). Patients with ALS-associated VAPB mutations indicated malfunctions in Ca^2+^ signaling and storage, excessive ER stress, and neuronal death as a result of inhibition of ATF6 (Ghemrawi and Khair, 2020). Furthermore, upregulation of PERK, IRE1α, and ATF6 was found in the ALS mouse models (Ghemrawi and Khair, 2020; Zhao et al., 2022).

### Targeting ER stress-induced apoptotic cell death

According to the critical role of ER stress in the occurrence of neuronal cell death in neurodegenerative diseases, targeting associated pathways seem to have hopeful effects on protecting neurons from death (Figure 1). Among three arms of UPR in ER stress conditions, the PERK pathway is the most well-studied in the neuroprotective effects of inhibition of ER stress. In parallel with this, Salubrinal, an anti-ER stress compound, has been well investigated in neurodegenerative disease pathology and treatment (Gupta S. et al., 2021; Ajoolabady et al., 2022). Salubrinal is an activator of UPR, which raises ER chaperone levels, including BiP. It inhibits eIF2α dephosphorylation which can attenuate neuronal death by interfering with death-related signaling pathways, including ATF4 or ASK1 (Figure 1, Table 2; Niso-Santano et al., 2011; Wu et al., 2014; Sprenkle et al., 2017). Accumulating evidence indicates that Salubrinal reduced ER accumulation of α-synuclein and significantly protected against α-synuclein-mediated dopaminergic (DA) neuronal death in transgenic mouse models (Colla et al., 2012). Also, Salubrinal reduced the accumulation of mutant huntingtin (mHTT) by upregulation of BiP and p-eIF2α, and prevent neuronal cell death (Maity et al., 2022). In addition, the drug Adaptaquin blocks Tribbles pseudokinase 3 (Trib3) induction by inhibiting ATF4 and CHOP activity probably through an eIF2α-independent mechanism, leading to neuronal protection in mouse models of PD (Figure 1, Table 2). More investigation is required for the neuroprotective effects of Adaptaquin in ER stress-induced neuronal cell death (Karuppagounder et al., 2016; Aime et al., 2020). Moreover, the PKR inhibitor “C16” can reduce transcriptional induction of pro-apoptotic target genes of ATF4, such as CHOP, Trib3, and PUMA (Figure 1, Table 2). This could significantly reduce MPP+ and 6-OHDA neurotoxin-induced neuronal cell death in PD models (Demmings et al., 2021). According to the experimental study, PUMA expression can be downregulated by directly targeting CHOP to decrease ER stress-induced neuronal apoptosis (Galehdar et al., 2010). Notably, pharmacological inhibition of ATF4, using imidazole-oxindole PKR inhibitor, indicated neuroprotection against neurotoxin-induced cell death in PD models (Demmings et al., 2021). Comparing motor neuron death in ATF4-ablated transgenic ALS mouse models with those expressing normal levels of ATF4 demonstrated the possible role of ATF4 ablation in neuroprotection against ALS by reducing apoptosis components, including CHOP (Matus et al., 2013). Likewise, another study revealed an increase in neuronal death in PD rat models by overexpression of ATF4 using recombinant Adeno-Associated Virus (rAAV; Gully et al., 2016). Halliday et al. (2017) revealed the inhibition of UPR-induced p-eIF2α signaling and neuronal survival by two chemical compounds termed “Trazodone” and “dibenzoylmethane” (DBM) in prion-infected mice (Figure 1, Table 2), presumably by reversing translational attenuation and lowering levels of ATF4 and CHOP which needs to be more inquired in other neurodegenerative diseases including AD and PD. Interestingly, it has been demonstrated that PERK inhibitor GSK2606414 (Figure 1, Table 2), despite its pancreatic toxicity (Halliday et al., 2015), inhibits and reduces PERK expression, which has a neuroprotective effect on DA neurons in Substantia Nigra pars compacta (SNpc) of PD mouse models, and improves the motor performance and neuronal excitability of PD mice (Mercado et al., 2018). In addition, inhibition of PERK signaling with IRSIB has been investigated in ALS rodent models, and a reduction in ATF4 and CHOP levels has been indicated (Figure 1, Table 2), which results in significant neuronal survival. In the same study, a reduction in IRE1-dependent signaling has also been indicated (Halliday et al., 2015, 2017).

**Table 2:** The function and target molecules of drugs tested in neurodegenerative disease models in different cellular stress conditions.

**Condition**	**Drug name**	**Target Molecule(s)**	**Function**	**Reference**
ER stress	Salubrinal	ATF4	Inhibit transcription of apoptotic factors	Kim et al. (2014) and Ghemrawi and Khair (2020)
	Salubrinal	ASK1	Prevent apoptosis by affecting downstream molecules of JNK/MAPK pathway	Ghemrawi and Khair (2020)
	Adaptaquin	ATF4	Inhibit transcription of apoptotic factors	Aime et al. (2020)
	C16	ATF4	Inhibit transcription of apoptotic factorsreduce neuronal death caused by neurotoxins	Demmings et al. (2021)
	Trazodone	p-eIF2α	Decrease ATF4 levels	Halliday et al. (2017)
	Dibenzoylmethane	p-eIF2α	Decrease ATF4 levels	Halliday et al. (2017)
	GSK2606414	PERK	Inhibit PERK pathway by preventing the phosphorylation of eIF2α	Mercado et al. (2018)
	IRSIB	PERK	Reduce ATF4 and CHOP levels	Halliday et al. (2015) and Halliday et al. (2017)
	KIRA6	IRE1	Break IRE1 oligomersinhibit RNase activity of IRE1	Ghosh et al. (2014)
	Kaempferol	ATF6, IRE1, PERK, CHOP	Reduce the expression of mentioned factors	Abdullah and Ravanan (2018)
	DHCR24	BiP, CHOP	Reduce the expression of mentioned factorsattenuate apoptotic signaling pathways	Lu X. et al. (2014)
	xestospongin C	IP3R	Regulate Ca2+ homeostasis	Wang et al. (2019)
	Ryanodine	RyR	Regulate Ca2+ homeostasis	Adasme et al. (2015)
	4-Phenyl Butyric acid	Unfolded protein	Interaction between hydrophobic regions of the chaperone and hydrophobic regions of the unfolded protein	Pao et al. (2021)
Oxidative stress	Humanin	Pro-apoptotic Bcl-2 family	Inhibit CytC and AIF release	Ma and Liu (2018) and Hazafa et al. (2021)
	L-NAT	Caspase	Inhibit CytC and AIF releaseinhibit caspase activity	Li et al. (2015) and Sirianni et al. (2015)
	NAS	NA	Increase antioxidant levels	Yoo et al. (2017)
	CoQ10	Mitochondrial permeability transition pore	Inhibit CytC and AIF release	Young et al. (2007) and Akanji et al. (2021)
	Diphenyleneiodonium	NADPH oxidase	Inhibit ROS production by NOX activity	Chocry and Leloup (2020)
	Apocynin	NADPH oxidase	Inhibit ROS production by NOX activity	Chocry and Leloup (2020)
	VAS2870	NADPH oxidase	Inhibit ROS production by NOX activity	Chocry and Leloup (2020)
	Aucubin	Nrf2	Regulating mitochondrial membrane potential and decreasing ROS generation	Wang et al. (2020) and Li Y. C. et al. (2021)
	Salidroside	Pro-apoptotic Bcl-2 family caspase	Inhibit CytC and AIF release inhibit caspase activation	Wang et al. (2015)
	Borneol	pro-apoptotic Bcl-2 family	Inhibit Cyt C and AIF release	Hur et al. (2013)
	[6]-Gingerol	Free radicals	Scavenge free radicals and decrease phospholipid peroxidation	Lee et al. (2011)
	Isoorientin	GSK-3β	Blocks GSK-3β *via* an ATP noncompetitive inhibition to attenuate tau hyperphosphorylation	Liang et al. (2016)
Neuroinflammation	Rosmarinic acid	miR-155-5p	Attenuate inflammation by miR-155–5p regulation	Lv et al. (2020)
	Alpha1-antitrypsin	Calpain	Inhibit calpain activity	Feng et al. (2020)
	Alpha1-antitrypsin	NA	Attenuate microglial inflammation	Feng et al. (2020)
	Epigallocatechin-3-gallate	NA	Attenuate neuroinflammation	Cheng C.-Y. et al. (2021)
	Aucubin	NF-κB, JNK, p38, and ERK	Reduce phosphorylation levels of mentioned factors to decrease inflammatory factor overexpression	Li Y. C. et al. (2021)
	Hesperetin	TLR4, NF-κB ERK, p38 MAPK	Modulate TLR4/NF-κB signaling pathway downregulate the phosphorylation of ERK and p38 MAPK	Jo et al. (2019) and Muhammad et al. (2019)
	15d-PGJ2	PPAR-_γ_	Inhibit production of interleukins	Xu et al. (2008)
	Anakinra	IL-1	Inhibit pyroptosis mediated by IL-1β	Wang et al. (2019)
	GW501516	PPAR-β/δ	Attenuate NLRP3-mediated neuroinflammation	Chen et al. (2019) and Altinoz et al. (2021)
	MCC950	NLRP3 inflammasome	Inhibit inflammasome activation	Gordon et al. (2018) and Deora et al. (2020)
	Dihydromyricetin (DHM)	NLRP3 inflammasome	Inhibit inflammasome activation	Feng et al. (2018)
	Benzyl isothiocyanate (BITC)	IL-1β, NLRP3 inflammasome	Inhibition of IL-1β release and NLRP3 inflammasome	Lee et al. (2016)
	Dopamine	Dopamine D1 receptor	The binding of cAMP with NLRP3 and NLRP3 degradation	Yan et al. (2015)
	Baicalein	NLRP3 inflammasome, Caspase	Decreasing pro-inflammatory cytokines production	Rui et al. (2020)
	Resveratrol	NF-κB	Decrease phosphorylation of NF-κB Inhibit microglial activation	(Zhong et al. (2012), Zhang et al. (2017), and Huang et al. (2021)

Inhibition of the IRE1 pathway is also a possible way to attenuate neuronal cell death. For instance, Kinase-Inhibiting RNase Attenuator 6 (KIRA6) inhibits apoptosis by breaking IRE1 oligomers and inhibiting RNase activity of IRE1α (Figure 1, Table 2; Ghosh et al., 2014). Given the vital role of ASK1 in IRE1-mediated UPR and inducing apoptosis, targeting and deletion of ASK1 in mutant SOD1-transgenic mice have been indicated to mitigate motor neuronal death (Homma et al., 2009). Additionally, evidence shows that overexpression of XBP1 protects DA neurons against neurotoxin-induced ER Stress-associated cell death (Valdes et al., 2014; Shi et al., 2022). Furthermore, upregulation of autophagy by targeting XBP1 in ALS and HD models is known to be another way of protection from neuronal cell death (Remondelli and Renna, 2017). The experiments have been demonstrated that ablation of ATF6 facilitates DA neuronal death caused by neurotoxins, including 6-hydroxydopamine (6-OHDA) and 1-methyl-4-phenyl-pyridinium (MPP+; Shi et al., 2022). This indicates the plausible role of the third arm of UPR pathways in inducing neuronal death. Kaempferol (Table 2), a plant-derived ER stress-induced cell death inhibitor, has also reduced the expression of ATF6, PERK, IRE1α, as well as CHOP in Brefeldin A (BFA)-induced ER stress in IMR32 cell lines. More investigations is needed to determine whether it is effective in animal and human neurodegenerative models (Abdullah and Ravanan, 2018). It is also claimed that 3β-Hydroxysteroid-Δ24 reductase (DHCR24) can protect neuronal cells by reducing BiP and CHOP levels and attenuating ER stress-specific apoptotic signaling pathways (Table 2; Lu X. et al., 2014). Targeting other indirect factors involved in ER stress, such as IP3 receptors and Ryanodine receptors, has also been examined. Remarkably, the first research confirming blocking Inositole triphosphate receptors (IP3Rs) and ryanodine receptors (RyRs) to decrease ER stress-induced Ca^2+^ dyshomeostasis in DA neurons revealed that a RyRs blocker (RY) markedly reduced 6-OHDA-induced cytosolic Ca^2+^ increases. In contrast, an IP3Rs blocker (Xes) had no considerable effect on cytosolic Ca^2+^ levels and neuronal cell death (Table 2). Moreover, pre-treatment with an ER stress inhibitor 4-phenyl butyric acid (4-PBA) had a neuroprotective effect on DA neurons from 6-OHDA-induced apoptosis (Table 2; Huang et al., 2017).

## Oxidative stress-induced apoptotic cell death in neurodegenerative diseases

### Mechanism of oxidative stress-induced apoptotic cell death

**Figure 2:**
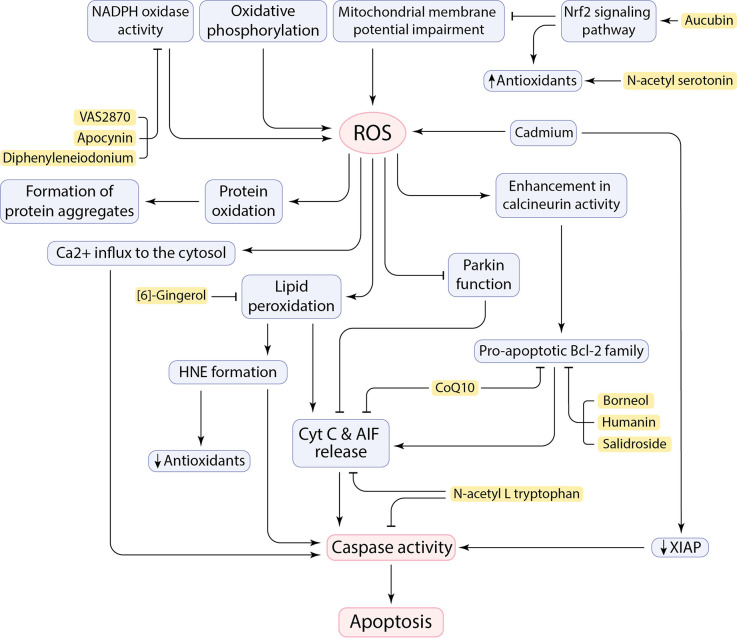
Cause and consequences of ROS production in the CNS and the neuroprotective effects of inhibitory factors (shown in yellow box).

Healthy mitochondria produce ROS as a byproduct of oxidative phosphorylation mainly as signaling messengers (Hajam et al., 2022; Trushina et al., 2022), while defective mitochondria generate aberrant amounts of ROS and cause oxidative stress and suspend cellular homeostasis due to the disruption of the balance between ROS generation and antioxidant function (Figure 2; Höhn et al., 2020; Holubiec et al., 2022). Neurons are susceptible to produce free radicals due to being metabolically very active. Evidently, any pathological situation or dysfunction in neurons can generate excess ROS leading to oxidative stress (Bhat et al., 2015). Given that the metabolism rate of neurons is very high, the brain has a high oxygen consumption rate (20%–25% of the total body oxygen consumption). Furthermore, the high content of easily peroxidizable unsaturated fatty acids (PUFA) and the relative paucity of antioxidant enzymes compared with other organs makes the brain vulnerable to free radical damage (Nunomura et al., 2007; Rocha et al., 2018). Therefore, the excessive production of ROS and RNS resulting from various factors, including calcium influx and mitochondrial dysfunction, can compromise cell fidelity and exacerbate disease progression. Hydrogen peroxide (H_2_O_2_), superoxide anion (O_2_^−^), and highly reactive hydroxyl radical (HO^•^) are the ROS involved in neurodegeneration. The RNS, such as nitric oxide (NO), are also found to have a deleterious effect on neurons (Singh et al., 2019; Korovesis et al., 2023).

ROS adversely affects the oxidation or peroxidation of specific macromolecules such as lipid peroxidation to malondialdehyde (MDA), protein carbonylation, and oxidation of specific nucleic acids (Singh et al., 2019). It has been claimed that ROS allows Cyt C and AIF to be released from the inner mitochondrial membrane (IMM) and initiate an apoptotic cascade (Figure 2; Bhat et al., 2015). The neural brain cells are enriched in PUFA, such as docosahexaenoic acid, arachidonic acid, and cardiolipin, which makes cells susceptible to lipid peroxidation and subsequent outcomes (Höhn et al., 2020; Falabella et al., 2021). For example, Cardiolipin (CL), a specific phospholipid of IMM, located in the sites of ROS production in the mitochondrial electron transport chain, can potentially be a target for ROS due to its high composition of unsaturated acyl chains. After peroxidation by ROS, CL is supposed to be involved in the conformational changes in IMM and the release of pro-apoptotic proteins, including Cyt C (Bhat et al., 2015; Falabella et al., 2021). However, a serine protease called HTRA2 takes part in the inhibition of pro-apoptotic protein release from mitochondria, but its function may not be sufficient, or it may be disturbed (Bhat et al., 2015). Moreover, the brain is also enriched in redox-active metals (copper and iron) that involve in generating free radicals and peroxidation of lipids (Sbodio et al., 2019; Falabella et al., 2021). These metals could be reduced by proteins such as Aβ, which leads to the formation of H_2_O_2_ and pro-apoptotic lipid peroxidation (LPO) products, such as 4-hydroxy-2-nonenal (HNE; Opazo et al., 2002; Jiang et al., 2009). In addition to the role of HNE in decreasing antioxidant levels by reacting with sulfhydryl groups (Taso et al., 2019), HNE forms stable adducts with amine or thiol groups in proteins and may eventually anomalously activate caspases and triggers neuronal cell death (Figure 2; Gaschler and Stockwell, 2017; Barrera et al., 2018). It has been demonstrated that Cadmium (Cd) could easily penetrate the blood-brain barrier and contribute to neurotoxicity. Cd can induce mitochondrial ROS production in neurons as well as downregulation of x-linked inhibitor of apoptosis (XIAP), leading to an increase in mouse double minute 2 (MDM2). Consequently, decrease in p53 facilitates neuronal apoptosis cell death (Figure 2; Zhao et al., 2020).

ROS can influence protein oxidation and contribute to the formation of insoluble protein aggregates (Figure 2), including Aβ peptides and NFTs, α-synuclein, and mSOD1. Oxidative stress can enhance expression of gamma-secretase and beta-secretase (BACE1) through activation of MAPK pathway and involves in Aβ production in neurons and amyloidogenic processing of amyloid precursor protein (APP; Tamagno et al., 2005; Lin and Beal, 2006; Höhn et al., 2020). Oxidative stress also increases tau phosphorylation by activation of glycogen synthase kinase 3 (GSK3; Lin and Beal, 2006). ROS also mediate JNK/stress-activated protein kinase pathways, which subsequently contributes to hyper-phosphorylation of tau proteins, formation of intracellular NFTs, and Aβ-induced neuronal death (Liu et al., 2017). Indeed, hydrogen peroxide and deficiency of mitochondrial antioxidant enzymes has been tested in animal models, which led to increase in Aβ levels (Gerakis and Hetz, 2019) and neuronal cell death (Lin and Beal, 2006). Interestingly, it has been revealed that neurons close to Aβ-plaques seem to be more at risk of cell death due to more severe toxicity of the microenvironment caused by oxidative stress in AD (Xie et al., 2013). Aβ-mediated oxidative stress can enhance the activity of a serine/threonine phosphatase, known as calcineurin, and promotes neuronal death by associating with caspases and/or triggering pro-apoptotic Bcl-2 proteins (Figure 2; Awasthi et al., 2005; Akanji et al., 2021). Moreover, ROS can have noxious effects by affecting Ca^2+^ cation channels on the ER and plasma membrane. Impaired Ca^2+^ channels can, in turn, lead to Ca^2+^ influx to the cytosol, as well as impairment in pumping intracellular Ca^2+^ out of the cell to maintain homeostasis (Brini et al., 2014). Toxic levels of calcium can trigger cell death through activation of apoptotic factors, including calcium-dependent proteases calpain and caspases (Figure 2; Fairless et al., 2014). Importantly, ROS has also a deleterious effect on nuclear factor erythroid 2-related factor 2 (Nrf2) regulation. Nrf2 is a transcription factor that has an essential role in regulating cellular redox homeostasis (Kovac et al., 2015). Reduced levels of Nrf2 can subsequently result in mitochondrial dysfunction and apoptosis. Therefore, upregulation of Nrf2 reduces oxidative stress by promoting the expression of antioxidant enzymes (Figure 2; Li Y. C. et al., 2021). Besides, the Repressor element 1-silencing transcription factor (REST) regulates cell death-associated genes, including BAX, BH3 interacting domain death agonist (BID), and also PUMA, and maintains resistance to stress conditions. REST-depleted neurons are more susceptible to oxidative stress and anomalously express apoptosis-inducing genes which facilitates neuronal death in AD (Lu T. et al., 2014). Remarkably, upregulation of the transient receptor potential melastatin-2 (TRPM2) in the SNpc of human PD brains agrees with the role of TRPM2 in ROS-induced cell death in PD pathogenesis (Malko et al., 2021). The function of Parkin can be affected by mitochondrial dysfunction and oxidative stress. This will promote Cyt C release and caspase-9 activation, which leads to neuronal cell death and facilitating PD pathogenesis (Figure 2; Lin and Beal, 2006). Defect in Complex I of the mitochondrial electron transport chain by aggregation of α-synuclein and PTEN-induced putative kinase 1 (PINK1) mutations can be also involved in PD pathogenesis by inducing neuronal apoptosis and failure in maintaining mitochondrial membrane potential, respectively (Liu et al., 2017; Morales-Martínez et al., 2022).

### Oxidative stress-associated alterations of apoptotic factors

Besides the oxidation of macromolecules is elevated in the brain of patients, decreased levels of antioxidants, including uric acid, vitamin C and E, superoxide dismutase (SOD), catalase, and especially the antioxidant glutathione (GSH), lead to decreased detoxification of ROS in the brain cells which has been discovered in various AD, PD, and other neurodegenerative disease patients (Singh et al., 2019). Oxidative damage occurs before the onset of significant plaque pathology in the AD by triggering glycogen synthase kinase 3 (Lin and Beal, 2006; Wu et al., 2019). Overproduction of ROS and RNS in AD patients has been detected. Additionally, 8-hydroxydeoxyguanosine (8-OHdG), a biomarker of oxidative damage, was elevated in AD ventricular CSF (Niedzielska et al., 2016). However, alterations in plasma levels of antioxidants in AD patients are paradoxical in experimental reports (Niedzielska et al., 2016). Aβ plaques can cause Ca^2+^ dyshomeostasis in ER leading to Ca^2+^ influx in the cytosol. Consequently, endogenous GSH levels are reduced, and ROS can cause neurotoxic effects (Liu et al., 2017). The alterations of transition metals, including Cu^2+^, Zn^2+^, Fe^3+^, have been assessed in AD samples. The results indicated that transition metals seem to be imbalanced in AD brains which contributed to oxidative damage and subsequent neuronal death (Bhat et al., 2015). Evidence also shows reduction in nuclear REST levels in neurons of degenerated regions in AD brains, such as prefrontal cortical and hippocampal neurons, which transcriptional dysregulation in apoptotic genes and vulnerability to oxidative stress has made them susceptible to apoptosis cell death (Lu T. et al., 2014). Elevated levels of activated JNK have been reported in post-mortem AD samples, which is probably associated with Aβ formation. JNK implicates in the upregulation of BACE1 and promotes the formation of Aβ, leading to oxidative stress and neuronal apoptosis cell death (Yao et al., 2005; Guglielmotto et al., 2011; Sbodio et al., 2019).

HNE levels were significantly high in the CSF of AD and PD patients, which can be considered as an important reason for neuronal demise and behavioral symptoms in neurodegenerative diseases (Taso et al., 2019). Decreased level of GSH in the Substantia Nigra (SN) of PD patients is one of the earliest biochemical alterations that facilitate the neurotoxic effects of ROS (Niedzielska et al., 2016). Moreover, overexpression of α-synuclein in transgenic mice results in mitochondrial dysfunction and increased oxidative stress (Song et al., 2004). It has been observed that aberrant activity of mutant human SOD1 in ALS patients leads to increase in the level of free radicals in CSF, serum, and urine samples of ALS patients, which exacerbates neuronal damage (Liu and Wang, 2017). Indeed, mSOD1 accumulation in outer mitochondrial membrane (OMM) can result in mitochondrial dysfunction and promotes aberrant ROS production. In an experimental study, mice expressing mSOD1 showed more oxidative damage to mitochondrial lipids and molecules (Mattiazzi et al., 2002; Liu et al., 2004). P53 can regulate genes involved in oxidative stress and mitochondrial function. Environmental toxicants such as bisphenol A (BPA) play a role in inducing neurotoxicity by significantly increased oxidative stress. BPA subsequently leads to upregulation in apoptotic inducing factors, including p53, PUMA, and Drp-1 (Ishtiaq et al., 2021). Additionally, in Huntington’s disease pathology, mHTT can interact with p53 and increase p53 levels, and eventually upregulates apoptotic factors BAX and PUMA, which leads to apoptosis (Bae et al., 2005; Lin and Beal, 2006). mHTT also interacts with mitochondrial membranes, causing mitochondrial abnormalities and an increase in ROS generation, which potentially leads to neuronal degeneration and cell death (Ross and Tabrizi, 2011; Liu et al., 2017). Elevated levels of lipid peroxidation and decreased levels of GSH content have been indicated in the plasma of HD patients (Klepac et al., 2007). In addition, increased levels of 8-OHdG have been observed in the serum of HD patients and post-mortem HD samples (Sbodio et al., 2019).

### Targeting oxidative stress-induced apoptosis cell death

Undoubtedly, oxidative stress has neurotoxic effects in the pathogenesis of neurodegenerative diseases. However, the exact molecular pathways remain unclear and need to be more inquired about finding a promising therapeutic strategy to decrease neuronal death in neurodegenerative diseases and extend the lifespan of patients. In this regard, the antioxidant properties of many candidate compounds have been reported. In addition, many other molecules that mediate oxidative stress-induced apoptosis have been targeted to prevent neuronal cell death and disease progression (Figure 2). A cytoprotective polypeptide called Humanin (HN), which is encoded by mtDNA, has neuroprotective activity against cellular stress conditions, such as oxidative stress. HN regulates mitochondrial function by targeting apoptotic factors and inhibits apoptosis by upregulation of Bcl-2 and downregulation of Bid and Bax (Figure 1, Table 2; Hazafa et al., 2021). Also, some amino acid derivatives have shown anti-apoptotic effects in ALS models *in vitro* (Sirianni et al., 2015). N-acetyl-L-tryptophan (L-NAT) and N-acetyl-DL-tryptophan (DL-NAT) have inhibited neuronal cell death in H_2_O_2_-induced NSC-34 motor neurons (Figure 1, Table 2). L-NAT inhibits the release of Cyt C/Smac/AIF from mitochondria, as well as inhibition of caspase activity, thereby preventing neuronal apoptosis cell death (Sirianni et al., 2015). Another study by Yoo et al. (2017) showed that N-acetyl serotonin (NAS) has anti-apoptotic properties by activating neurotrophic signaling TrkB/CREB/BDNF pathways. NAS induces and activates the expression of antioxidant enzymes to reduce the level of ROS (Figure 1, Table 2). It also regulates anti- and pro-apoptotic factors and restores mitochondrial membrane potential to prevent neuronal cell death in neurodegenerative disease models (Yoo et al., 2017). According to evidence, mitochondrial permeability transition pore (mPTP) can increase mitochondrial calcium retention and cause cell death. CoQ10 is considered as an inhibitor of mitochondrial permeability transition pore and protects neurons from oxidative stress and apoptosis. The exact protective mechanism of CoQ10 is still indistinct and needs more experiments. CoQ10 may decrease apoptosis by maintaining the integrity of the mitochondrial membrane and inhibiting Cyt C release. CoQ10 may also decrease the Bcl-2 protein level and prevent caspase activation (Figure 1, Table 2; Akanji et al., 2021). As previously mentioned, protein aggregates can potentially induce oxidative stress in neuronal cells. For instance, Aβ can interact and bind to Aβ-binding alcohol dehydrogenase (ABAD), a mitochondrial-matrix protein, and induce apoptosis and free-radical generation. Blocking the interaction of Aβ and ABAD with a “decoy peptide” suppress oxidative stress and neuronal death. In contrast overexpression of ABAD in mouse models contribute to exaggerating cellular stress and further complications (Lustbader et al., 2004). Moreover, NADPH oxidase (NOX) catalyzes the formation of O_2_^−^ and participates in elevating neurotoxicity and increasing cell death in HD. Treatment of HD models with NOX inhibitors, including diphenyleneiodonium, apocynin, and VAS2870, prevented neurons from cell death (Figure 1, Table 2; Sbodio et al., 2019).

In several studies, it has been reported that plant iridoids have therapeutic applications in several neurodegenerative diseases by regulating apoptotic factors and neuroprotective proteins (Dinda et al., 2019). Aucubin (AU) is an iridoid glycoside with neuroprotective properties, which significantly increases cell viability in neurons via oxidative stress reduction. AU enhances the antioxidant capacity of cells through the Nrf2 signaling pathway and decreases ROS-induced neuronal apoptosis by regulating mitochondrial membrane potential and reducing ROS generation (Figure 1, Table 2; Li Y. C. et al., 2021). The pharmacological effects of other herbal compounds have also been investigated. Salidroside (Sald), is a Chinese plant-derivative compound that could detoxify neurons by suppressing the elevation of the intracellular ROS level and induction of antioxidant enzymes. Sald also participates in the downregulation of pro-apoptotic protein Bax and upregulation of anti-apoptotic protein Bcl-xl, and prevents neuronal cell death (Figure 1, Table 2; Zhang et al., 2010). Some plant-derived organic oils, including a bicyclic monoterpene termed “Borneol”, indicated neuroprotective effects against H_2_O_2_-induced apoptosis *in vitro*. Borneol alleviates neuronal apoptosis by inhibiting Cyt C and AIF release through increase in the expression of anti-apoptotic protein Bcl-2 and decrease in expression of pro-apoptotic protein Bax (Figure 1, Table 2; Hur et al., 2013). -gingerol also attenuates Aβ-induced oxidative stress. Studies revealed that -gingerol scavenges free radicals and decreases phospholipid peroxidation, as well as improves cellular redox balance (Figure 1, Table 2; Lee et al., 2011). Increase in the activity of glycogen synthase kinase-3β (GSK-3β) under oxidative stress condition leads to Nrf2 dysregulation (Kumar et al., 2012). Thus, GSK-3β inhibitors, including an anti-oxidative phytochemical known as Isoorientin, can have neuroprotection against oxidative damage by regulating Nrf2 antioxidant activity (Figure 1, Table 2; Lim et al., 2007; Gianferrara et al., 2022).

## Neuroinflammation-induced cell death in neurodegenerative diseases

### Mechanism of neuroinflammation-induced cell death

**Figure 3:**
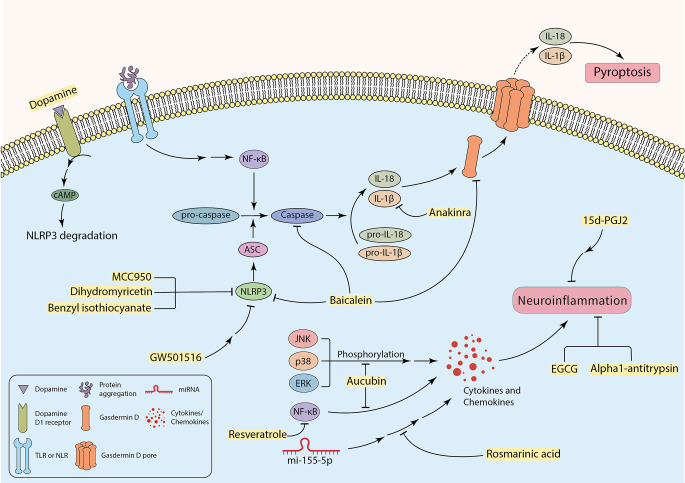
The possible role of inhibiting inflammatory factors to attenuate neuroinflammation-induced neuronal cell death.

Research findings have indicated that several neurodegenerative diseases are associated with inflammation (Kwon and Koh, 2020). Activated microglia and T lymphocytes have been detected in the SN of PD patients (Dias et al., 2013). In parallel with this, high expression levels of chemokines, interleukins, interferons, and tumor necrosis factor-α (TNF-α) have been discovered in the striatum and substantia nigra of PD post-mortem brain samples and CSF of AD patients (Hirsch and Hunot, 2009; Llano et al., 2012; Gelders et al., 2018). A neurotoxic microenvironment can be promoted by the continuous secretion of inflammatory mediators from microglia and astrocytes, thus facilitating neural degeneration, and glial cell death (Pardillo-Díaz et al., 2022; Song et al., 2022). In addition to apoptosis cell death, pyroptosis, a non-apoptotic programed cell death, can also occur in the CNS, which is mainly mediated by inflammatory processes. Pyroptosis is characterized by cell swelling, formation of pores in the plasma membrane carried out by cleaved Gasdermin D, and the release of pro-inflammatory cytosolic contents into the extracellular space (Figure 3; Walle and Lamkanfi, 2016; Man et al., 2017; Wang et al., 2019). Besides, some specific caspases, including caspase-1, 4, 5, 11, are called “inflammatory caspases”, can mediate pyroptosis (Taylor et al., 2008; Gaidt and Hornung, 2016). Some factors are associated with initiation of inflammatory cascades and promotion of disease pathology. For instance, ER stress can cause inflammation in neurodegenerative diseases. In other words, inducing ER stress in neurons mostly initiates apoptosis, whereas intense ER stress in glial cells can potentially trigger inflammation in neurodegenerative diseases (Sprenkle et al., 2017). The UPR can increase the production and the release of inflammatory factors, such as transcription factor “nuclear factor kappa-light-chain-enhancer of activated B cells” (NF-κB), interleukin 1 (IL-1), IL-6, IL-8, and TNF-α (Feng et al., 2017). Furthermore, in ER stress condition, p-IRE1 can bind to the TRAF-2 protein and forms the TRAF2-IRE1 complex. The complex may bind to ASK-1 and activate the JNK signaling pathway that enhances inflammation (Vukic et al., 2009; Mohammed-Ali et al., 2015). In addition, p-PERK also facilitates neuroinflammation by inducing the JAK1/STAT3 signaling pathway in glial cells (Meares et al., 2014). Moreover, neuroinflammation is a cause and a consequence of chronic oxidative stress. Studies indicate that the production of free radicals (such as ROS) are elevated in neurodegenerative diseases, which can be due to neuroinflammation (Dias et al., 2013; González-reyes et al., 2017). On the other hand, increased levels of ROS can contribute to pro-inflammatory gene transcription and release of cytokines, including IL-1, IL-6, and TNF-α (Sochocka et al., 2013; Teleanu et al., 2022). Debris of dead neurons may trigger glia-mediated neuroinflammation and initiate a pro-inflammatory cascade that can exacerbate disease progression (Wang et al., 2015; Joshi et al., 2019). Recently, it was revealed that microglial pro-inflammatory cytokines are associated with increased α-synuclein aggregation (Guo et al., 2020). Thereby, protein aggregates can be involved in inducing neuroinflammation in the CNS parenchyma.

It has been investigated that the failure to clear apoptotic cells and activation of glial cells by DAMPs and PAMPs can enhance inflammation response and subsequent neuronal damage and loss (Imbeault et al., 2014; Salter and Stevens, 2017; Voet et al., 2019; Cheng Y. et al., 2021). Activation of complex signaling cascades such as the NLR family pyrin domain containing 3 (NLRP3) inflammasome can be triggered by a wide range of factors including cellular stress, infection (Bader and Winklhofer, 2020; Mahboubi Mehrabani et al., 2022), protein aggregates, and activated microglia (Nichols et al., 2019; Bader and Winklhofer, 2020; Tansey et al., 2022). This phenomenon contributes to producing more neurotoxic cytokines and chemokines such as IL-1β, IL-6, TNF-α, and CCL2 (also known as monocyte chemo-attractant protein-1 or MCP-1), that cause enhancement in neurotoxicity and cell death (Sprenkle et al., 2017; Rocha et al., 2018; Joshi et al., 2019; Nichols et al., 2019). The exact mechanism of neuronal death through activation of NLRP3 inflammasome in microglia has not been perfectly discovered yet (Lee et al., 2019). In fact, NLRP3 inflammasome induces heteromer formation or aggregation of apoptosis-associated speck-like protein containing a caspase recruitment domain (ASC), which subsequently activates caspase-1. It results in the maturation of IL-1β and induction of pyroptosis using cleaved Gasdermin D and membrane pores (Figure 3; Stancu et al., 2019; Wang et al., 2019; Bader and Winklhofer, 2020; Feng et al., 2020; Onyango et al., 2021).

Specific receptors, including TLRs and NLRs expressing on neurons or neuroglia, can recognize extracellular neurotoxic protein aggregates (Nichols et al., 2019; Leng and Edison, 2021; Heidari et al., 2022). This may facilitate neuronal cell death (Li Y. et al., 2021) by involvement in caspase activation and secretion of pro-inflammatory factors through NF-κB activation (Figure 3; Rocha et al., 2018; Leng and Edison, 2021). The expression of the receptors for cytokines was indicated in DA neurons, making neurons more susceptible to damage and death (Hirsch and Hunot, 2009). Complement receptors and Fc receptors on microglia can also mediate pro-inflammatory responses independent from extracellular protein aggregates (Leng and Edison, 2021). Activation of microglia can potentially contribute to activating astrocytes, which rapidly upregulates inflammatory signaling molecules (Sims et al., 2022), and can potentially initiate or enhance nitrosative stress due to producing NO (Rocha et al., 2018). In support of this claim, the presence of activated astrocytes is confirmed in post-mortem brain samples of various neurodegenerative disease patients (Hashioka et al., 2021). DAMPs released by dying neurons may also activate microglia through the ionotropic P2X and metabotropic P2Y purinergic receptors and initiate an inflammatory response. For example, under pathological conditions, P2X7 receptors can be overexpressed in the CNS. ATP acts as a DAMP and activates P2X7 receptors, and promotes chronic inflammatory neurological disorders (Thawkar and Kaur, 2019). Post-mortem brain samples of AD patients showed overexpression of P2X7 receptors, which can be associated with disease pathology and progression. It is also suggested that P2X4 receptor overstimulation may result in neuronal cell death (Thawkar and Kaur, 2019). Interestingly, upregulation of P2X and P2Y receptors in ALS patients can subsequently lead to overproduction of TNF-α and cyclooxygenase-2 (COX2), which facilitates neurotoxicity (Liu and Wang, 2017).

### Targeting neuroinflammation-induced cell death

Even though the neuroinflammatory cascades exacerbate neurodegenerative disease progression and participate in neuronal death, the exact mechanism of this involvement and therapeutic strategies regarding targeting neuroinflammation is not well studied. According to some investigations, targeting neuroinflammation can be a promising therapeutic approach to decrease inflammation and its further complications in neurodegenerative diseases (Figure 3). Therefore, it can improve patients’ neuronal function in mental and physical activities. Incipiently, some specific microRNAs, such as miR-155-5p, can be key regulators of inflammatory cascades in neurodegenerative diseases. Overexpression of miR-155-5p has been reported in the CSF of AD and MS patients (Lv et al., 2020). It has been recently found that Rosmarinic acid (RA) can inhibit neuroinflammation in neurodegenerative disease samples by regulating miR-155-5p, leading to attenuation in inflammation-associated neuronal damage and loss (Figure 3, Table 2; Lv et al., 2020). Calpains as non-caspase proteases participate in the execution of neuronal cell death and cooperate with key factors of neuronal cell death. Thus, targeting these proteases may result in neuroprotection. Studies have demonstrated that Alpha1-antitrypsin (A1AT) can attenuate microglial neuroinflammation as well as inhibition of calpain activity (Figure 3, Table 2; Feng et al., 2020). Other novel strategies have also been recruited with a focus on neuroinflammation. A recent study by Cheng C.-Y. et al. (2021) targeted inflammation in the substantia nigra of lipopolysaccharide (LPS)-treated rats by liposomes carrying Epigallocatechin-3-gallate (EGCG), a natural antioxidant in green tea. It demonstrated neuroprotection by inhibiting neuroinflammation (Figure 3, Table 2; Cheng C.-Y. et al., 2021). Aucubin, which showed neuroprotective effects in oxidative stress-induced neurotoxicity, can also reduce phosphorylation levels of NF-κB, JNK, p38, and ERK, leading to a decrease in the level of inflammatory factors (Figure 3, Table 2; Li Y. C. et al., 2021). Additionally, a polyphenol named Resveratrol indicated a similar effect by down-regulation of the transcription factor NF-κB *in vitro* (Figure 3, Table 2; Zhong et al., 2012; Zhang et al., 2017). Targeting TLR4/NF-κB signaling pathway by Hesperetin, a Citrus flavonoid, protected neurons from neuroinflammation and apoptosis (Table 2; Muhammad et al., 2019). Many other similar signaling pathways can be inhibited, aiming to alleviate neuroinflammation in the CNS (Hou et al., 2021). Furthermore, targeting inflammation-associated receptors expressed on neurons can also be an approach. For example, 15d-PGJ2 is a peroxisome proliferator-activated receptor-gamma (PPAR-γ) agonist that inhibits the production of some interleukins and suppresses inflammation in microglial cells *in vitro* (Figure 3, Table 2; Xu et al., 2008). Suppressing neuroinflammation by targeting P2X7R has been studied, but blood-brain barrier (BBB) permeability limits candidate drugs, so more studies are needed in this case (Thawkar and Kaur, 2019). Intriguingly, Anakinra, an IL-1 receptor antagonist (Mahboubi Mehrabani et al., 2022), reaches CNS easily and inhibits the activity of IL-1β by binding to its receptor and mitigate pyroptosis in neurons (Figure 3, Table 2; Wang et al., 2019).

As mentioned in the former section, since the NLRP3 inflammasome plays a significant role in the enhancement of neurotoxicity, inhibition of NLRP3 and its subsequent pathways might be an effective method to decrease neuroinflammation-induced cell death. MCC950 is a small-molecule NLRP3 inhibitor that has inhibited inflammasome activation in rodent PD models leading to substantial neuroprotection, mitigation in motor deficits, and accumulation of α-synuclein aggregates (Figure 3, Table 2; Gordon et al., 2018). Noteworthy, the neurotransmitter dopamine can bind to the dopamine D1 receptor, which results in ubiquitination and degradation of NLRP3 via the binding of cAMP with NLRP3, leading to the restriction of NLRP3 activation (Figure 3, Table 2; Yan et al., 2015). Some experiments revealed attenuation of NLRP3-mediated neuroinflammation in PD mouse models using peroxisome proliferator-activated receptor beta/delta (PPAR-β/δ) agonist GW501516 (Figure 3, Table 2). There were some limitations with this drug, such as the resistance of the BBB to pass the drug to reach the brain parenchyma. Hence, this compound cannot be considered a candidate for PD treatment until the problem is not solved (Chen et al., 2019). A flavonoid derived from the roots of *Scutellaria baicalensis Georgi*, termed “Baicalein”, indicated anti-inflammatory and anti-pyroptosis properties in animal models of PD. Experiments suggest that Baicalein may play a role in preventing the loss of DA neurons by reducing the production of various pro-inflammatory cytokines. It can also inhibit NLRP3 and caspase-1 activation, and simultaneously suppress pyroptosis by targeting Gasdermin D in 1-methyl-4phenyl-1,2,3,6-tetrahydropyridine (MPTP) Induced Mice Model of PD (Figure 3, Table 2; Rui et al., 2020). Benzyl isothiocyanate (BITC) and dihydromyricetin (DHM) are other plant-derived compounds with anti-inflammation properties (Lee et al., 2016; Feng et al., 2018). BITC seems to have the neuroprotective effects by inhibition of IL-1β release and NLRP3 inflammasome inhibition in the BV2 microglial cells (Lee et al., 2016). Treatment of APP/PS1 transgenic mice with DHM improved neuroinflammation and memory function, as a result of decreased NLRP3 inflammasome activation (Figure 3, Table 2; Feng et al., 2018). Taken together, the detrimental effects of neuroinflammation in induced neuronal cell death must not be underestimated, and more research is required to provide a better understanding of mechanisms and underlying therapeutic strategies.

## Conclusion

As described elaborately, neuronal cell death plays a key role in demonstrating neurodegenerative disease manifestations. Understanding the exact mechanisms and pathways leading to cell death would provide the opportunity for researchers to recommend high-efficiency neuroprotective agents. Until now, many pre-clinical studies have been done in an attempt to cure neurodegenerative diseases, targeting crucial agents involved in well-known pathways leading to neuronal cell death. The field of targeting ER stress and UPR as a therapeutic approach to treat neurodegeneration is growing and has revealed considerable results. On the other hand, ROS damage to mitochondria and homeostasis of the neuron is prominent in neurodegenerative diseases. Hence, this has led to therapeutic approaches using agents with antioxidant properties or inducing the antioxidant activity of the neuron, resulting in inhibition of ROS-mediated neuronal injury. Activation of neuroglia and initiation of neuroinflammation could also lead to a neurotoxic microenvironment for neurons. Unfortunately, the exact mechanism of neuroinflammation-induced cell death is still under debate. Thus, there are not sufficient experimental results of targeting key components of neuroinflammation to decrease neuronal loss in neurodegenerative diseases directly. However, there is strong evidence implicating the role of inhibiting neuroinflammation in attenuating ROS- and ER stress-induced neuronal cell death. Nowadays, the focus on the neuroprotective effects of phytochemicals has significantly increased; nevertheless, there is still much to research and discover to approve phytochemicals as a promising therapeutic agent. Taken together, despite advances in the field of targeting cell death to treat neurodegenerative diseases, there is not an approved compound to directly inhibit cell death yet, so it needs intensive research to find a novel therapeutic strategy for treatment of neurodegenerative diseases.

## Author contributions

MK, FSH, and MM provided the idea and mainly wrote the manuscript. MK and AA contributed to the search and assessment of the available literature. MK designed and illustrated the figures. All authors contributed to the article and approved the submitted version.

## Figures and Tables

**Table:** 

4-PBA	4-phenyl butyric acid
6-OHDA	6-hydroxydopamine
8-OHdG	8-hydroxydeoxyguanosine
A1AT	alpha1-antitrypsin
ABAD	amyloid beta-binding alcohol dehydrogenase
AD	Alzheimer’s disease
AIF	apoptosis-inducing factor
ALS	amyotrophic lateral sclerosis
APP	amyloid precursor protein
ASC	apoptosis-associated speck-like protein containing a caspase activation and recruitment domain
ASK1	apoptotic signaling kinase 1
ATF4	activating transcription factor 4
ATF6	activating transcription factor 6
AU	aucubin
Aβ	amyloid-beta
BACE1	beta secretase 1
BAX	bcl2-associated X protein
BBB	blood-brain barrier
BFA	brefeldin A
BID	BH3 interacting domain death agonist
BiP	immunoglobulin binding protein
BITC	benzyl isothiocyanate
BPA	bisphenol A
CCL2	monocyte chemoattractant protein-1
Cd	cadmium
CHOP	C/EBP homologous protein
CL	cardiolipin
CNS	central nervous system
COX	cyclooxygenase
CSF	cerebrospinal fluid
Cyt C	cytochrome c
DA	dopaminergic
DAMP	damage-associated molecular pattern
DBM	dibenzoylmethane
DHCR24	3-beta-hydroxysteroid delta-24-reductase
DHM	dihydromyricetin
DL-NAT	N-acetyl-DL-tryptophan
DR5	Death Receptor 5
EGCG	Epigallocatechin-3-gallate
eIF2α	eukaryotic translation initiation factor 2 α
ER	endoplasmic reticulum
ERAD	ER-associated degradation
ERO1	ER Oxidase 1
GLP-1R	glucagon-like peptide-1 receptor
GRP78	78kDa glucose-regulated protein
GSH	glutathione
GSK-3β	glycogen synthase kinase-3beta
GSK3	glycogen synthase kinase 3
H2O2	hydrogen peroxide
HD	Huntington’s disease
HN	humanin
HNE	4-hydroxy-2-nonenal
HO•	hydroxyl radical
IL	interleukin
IMM	inner mitochondrial membrane
IP3R	inositol triphosphate receptor
IRE1α	inositol-requiring transmembrane kinase/endoribonuclease 1 α
JAK-STAT	janus kinase-signal transducer and activator of transcription
JNK	c-Jun N-terminal kinase
KIRA6	kinase-inhibiting RNase attenuator 6
L-NAT	N-acetyl-L-tryptophan
LPO	lipid peroxidation
LPS	lipopolysaccharide
MDA	malondialdehyde
MDM2	mouse double minute 2
mHTT	mutant huntingtin protein
MPP+	1-methyl-4-phenyl-pyridinium
MPTP	1-methyl-4phenyl1,2,3,6-tetrahydropyridine
mPTP	mitochondrial permeability transition pore
mSOD1	mutant superoxide dismutase 1
mtDNA	mitochondrial DNA
NAS	N-acetyl serotonin
NF-κB	nuclear factor kappa-light-chain-enhancer of activated B cells
NFT	neurofibrillary tangle
NLRP3	NLR family pyrin domain containing 3
NO	nitric oxide
NOX	NADPH oxidase
Nrf2	nuclear factor erythroid 2-related factor 2
O2-	superoxide anion
OMM	outer mitochondrial membrane
p38 MAPK	p38 mitogen-activated protein kinase
PAMP	pathogen-associated molecular pattern
PARK7	parkinsonism associated deglycase
PD	Parkinson’s disease
PERK	PKR-like ER kinase
PINK1	PTEN-induced putative kinase 1
PPAR	peroxisome proliferator-activated receptor
PUFA	polyunsaturated fatty acid
PUMA	p53-upregulated modulator of apoptosis
RA	rosmarinic acid
rAAV	recombinant adeno-associated virus
REST	repressor element 1-silencing transcription factor
RIDD	regulated IRE1α-dependent decay
RNS	reactive nitrogen species
ROS	reactive oxygen species
RY	ryanodine
RYR	ryanodine receptor
Sald	salidroside
SN	substantia nigra
SNpc	substantia nigra pars compacta
SOD1	superoxide dismutase
TNF	tumor necrosis factor
TRAF2	TNF receptor-associated factor 2
Trib3	tribbles pseudokinase 3
TRPM2	transient receptor potential melastatin-2
UPR	unfolded protein response
VAPB	vesicle-associated membrane protein-associated protein B
XBP1	X-box binding protein 1
XIAP	x-linked inhibitor of apoptosis
